# Effect of Support and Polymer Modifier on the Catalytic Performance of Supported Palladium Catalysts in Hydrogenation

**DOI:** 10.3390/molecules30183820

**Published:** 2025-09-19

**Authors:** Assemgul S. Auyezkhanova, Eldar T. Talgatov, Sandugash N. Akhmetova, Aigul I. Jumekeyeva, Akzhol A. Naizabayev, Aigul T. Zamanbekova, Makpal K. Malgazhdarova

**Affiliations:** D.V. Sokolsky Institute of Fuel, Catalysis, and Electrochemistry, Kunaev Str. 142, Almaty 050010, Kazakhstan; e.talgatov@ifce.kz (E.T.T.); s.akhmetova@ifce.kz (S.N.A.); a.dzhumekeeva@ifce.kz (A.I.J.); a.naizabayev@ifce.kz (A.A.N.); a.zamanbekova@ifce.kz (A.T.Z.); m.malgazhdarova@ifce.kz (M.K.M.)

**Keywords:** heterogeneous palladium catalysts, poly(4-vinylpyridine), chitosan, hydrogenation, 2-propen-1-ol, phenylacetylene, 2-hexyn-1-ol

## Abstract

In this study, we investigated the influence of polymer nature and support characteristics on the performance of Pd-based heterogeneous catalysts. Catalysts were prepared via sequential adsorption of poly(4-vinylpyridine) (P4VP) or chitosan (CS) and palladium ions onto MgO and SBA-15 supports under ambient conditions. The resulting hybrid materials were characterized by IR spectroscopy, scanning electron microscopy (SEM), transmission electron microscopy (TEM), and X-ray photoelectron spectra (XPS). TEM analysis revealed that Pd nanoparticles with an average size of 2–3 nm were well-dispersed on P4VP/MgO, while larger and less uniformly distributed particles (8–10 nm) were observed on SBA-15-based systems. Catalytic tests in the hydrogenation of 2-propen-1-ol, phenylacetylene, and 2-hexyn-1-ol under mild conditions (40 °C, 1 atm H_2_, ethanol) demonstrated that both the support and polymer type significantly influence activity and selectivity. P4VP-modified catalysts outperformed CS-containing analogs in all reactions. MgO-based systems showed higher activity and selectivity in 2-propen-1-ol hydrogenation compared to SBA-15-based catalysts. The 1%Pd–P4VP/MgO catalyst exhibited the best performance, with a reaction rate of 5.2 × 10^−6^ mol/s, 83.4% selectivity to propanol, and stable activity over 30 consecutive runs. In phenylacetylene and 2-hexyn-1-ol hydrogenation, all catalysts showed high selectivity to styrene (93–95%) and cis-2-hexen-1-ol (96–97%), respectively. The incorporation of P4VP polymer into the Pd/MgO catalyst leads to smaller and better-distributed palladium particles, resulting in enhanced catalytic activity and stability during hydrogenation reactions. These results confirm that the choice of polymer modifier and inorganic support must be tailored to the specific reaction, enabling the design of highly active and selective polymer-modified Pd catalysts for selective hydrogenation processes under mild conditions.

## 1. Introduction

Heterogeneous catalysts are widely used in various industrial catalytic processes [1,2,3,4,5]. Their advantages include mechanical strength, thermal and chemical stability, ease of recycling, and separation from reaction mixtures [2,4,6]. However, a significant disadvantage of heterogeneous catalysts is lower selectivity compared to homogeneous systems. This limitation has prompted researchers to develop new catalytic materials that combine the desired properties of both homogeneous and heterogeneous catalysts [7]. In this context, the selection of a suitable support material plays a critical role in determining the catalyst’s overall performance and efficiency. Typically, metal oxides, aluminosilicates, carbon are widely used as a solid support for metal catalysts due to their high thermochemical stability [2,8,9]. There are many studies on the use of polymers as supports for metal particles [3,9,10,11]. One of the main problems of using polymers as supports in catalytic systems is their low specific surface area, which limits the availability of active centers and, consequently, the efficiency of catalysis. To solve this problem, hybrid supports combining the structural advantages of mineral materials and the functionality of polymers are used.

Fixation of metal complexes with macromolecular ligands on inorganic supports is the focus of attention as a promising approach to the creation of heterogeneous catalysts with embedded metal nanoparticles [12,13,14]. Wissing et al. [15] developed Au and Pd nanoparticles (NPs) on zeolite L crystals decorated with photoactive polymer brushes. The role of the polymer is to reduce metal salts to the corresponding metallic NPs by releasing highly reductive ketyl radicals upon UV light irradiation, stabilizing the in situ formed metallic NPs and preventing their aggregation. Zeolite-polymer NPs were investigated in the stereoselective semi-hydrogenation of alkynes to Z-alkenes and oxidation of benzyl alcohols to aldehydes. Zhao et al. [16] employed chitosan (CS) and montmorillonite (Mt)-based composites, modified with either Al or Al–Fe, as supports for immobilizing Pd nanoparticles. Chitosan and Pd nanoparticles were incorporated into the interlayer spaces of the pillared Mt. The developed catalysts (Pd@CS/Al-Mt and Pd@CS/Al-Fe-Mt) showed high catalytic efficiency and stability in Sonogashira coupling reactions. Almost 100% yield was obtained in the Sonogashira reaction of iodobenzene with phenylacetylene at 0.16 mol% Pd loading for only 1 h. Nagarjuna et al. [17] synthesized a palladium catalyst using functionalization of polyethyleneimine (PEI) on alumina followed by adsorption of palladium. XPS analysis of the developed catalysts (Pd-PEI-AO and rPd-PEI-AO) showed favorable interaction of PdCl_4_^2−^ with PEI nitrogen molecules. The catalysts were successfully tested in the catalytic reduction of 4-nitrophenol and hydrogen generation from ammonia borane. Earlier, Rakap et al. [18] investigated the preparation and catalytic properties of polyvinyl butyral-immobilized palladium catalyst deposited on TiO_2_ (Pd-PVB-TiO_2_) in the hydrolysis of unstimulated ammonia-borane solution for hydrogen production. Maximum hydrogen generation rates of ~642 mL H_2_ min^−1^ (g Pd)^−1^ and ~4367 mL H_2_ min^−1^ (g Pd)^−1^ were observed during hydrolysis at 25 °C and 55 ± 0.5 °C, respectively. Szubiakiewicz et al. [19] reported that modification of silica with polyvinylpyrrolidone followed by loading of palladium catalyst improved the formation of the target product (tetrahydrofurfuryl alcohol) in the hydrogenation of furfural. It was shown that PVP promotes stabilization of Pd particles and prevents their agglomeration on the catalyst surface. Antonetti et al. [20] developed a polyketone-SiO_2_ (PK/SiO_2_) composite for use as a support for Pd catalysts. The synthesized catalysts were tested in the selective hydrogenation of cinnamaldehyde to hydrocinnamaldehyde in decalin and the oxidation of 1-phenylethanol to acetophenone in water. Pd/PK and Pd/SiO_2_ were also prepared for comparison. The Pd nanoparticles supported on the hybrid composite exhibited significantly smaller sizes and a more uniform size distribution compared to those supported on conventional SiO_2_ or PK. The PK/SiO_2_-based palladium catalysts demonstrated higher activity and stability. The authors attribute this to the improved surface area of the hybrid composite and the stabilizing effect of the polymer. Ródenas et al. [14] developed UVM-7/polydopamine (PDA)-based Pd nanoparticle catalysts supported on porous silica for the hydrogenation of 4-nitrophenol. The catalysts demonstrated higher efficiency compared to analogous systems lacking PDA.

In our previous works, we described a one-pot method for the synthesis of applied polymer–metal catalysts from aqueous solutions of polymers and metals. The method of preparation of such catalysts excludes the stages of high-temperature calcination and reduction [12,13,21,22].

The aim of the present work was to determine the effect of a polymer-modified and support on catalytic properties of palladium catalysts. Poly(4-vinylpyridine) (P4VP) and chitosan (CS) were used as the polymer modifier. The polymer–metal complexes were deposited on solid supports (MgO, SBA-15). The obtained results were confirmed by IR spectroscopy, TEM, XPS, SEM, EDX methods. To investigate the activity and selectivity of palladium catalysts, a set of unsaturated substrates (2-propen-1-ol, 2-hexyn-1-ol, and phenylacetylene) was selected to represent structurally diverse alkenes and alkynes bearing hydroxyl or aryl functional groups. This allows for a systematic evaluation of hydrogenation behavior across different functionalities [23]. Phenylacetylene serves as a key industrially relevant substrate due to its role as a styrene impurity that must be selectively removed via semi-hydrogenation [24]. 2-Hexyn-1-ol represents hydroxyl-containing acetylenic alcohols used in fine chemical synthesis, while 2-propen-1-ol is a common model compound for studying chemoselective hydrogenation of C=C bonds in the presence of polar groups [25,26]. Together, these substrates enable comprehensive insight into catalyst performance across a range of functionalized unsaturated molecules.

## 2. Results and Discussion

### 2.1. Characterization of Pd Catalysts Deposited on Polymer-Modified Supports

The palladium catalysts supported on polymer-modified supports were prepared by adsorption method. Table 1 summarizes the results of Pd^2+^ ions adsorption on MgO and silica (SBA-15), modified with poly(4-vinylpyridine) (P4VP) and chitosan (CS). The palladium content in the catalysts was estimated based on the change in the concentration of Pd^2+^ ions in the mother liquor before and after immobilization. The concentration of palladium in the solution was determined using photoelectric colorimetry (PEC). According to PEC data, palladium was almost completely adsorbed onto the surface of the supports. The calculated palladium content based on PEC measurements was approximately 1 wt.%, which was confirmed by energy-dispersive X-ray spectroscopy (EDX) elemental analysis.

**Figure 1:**
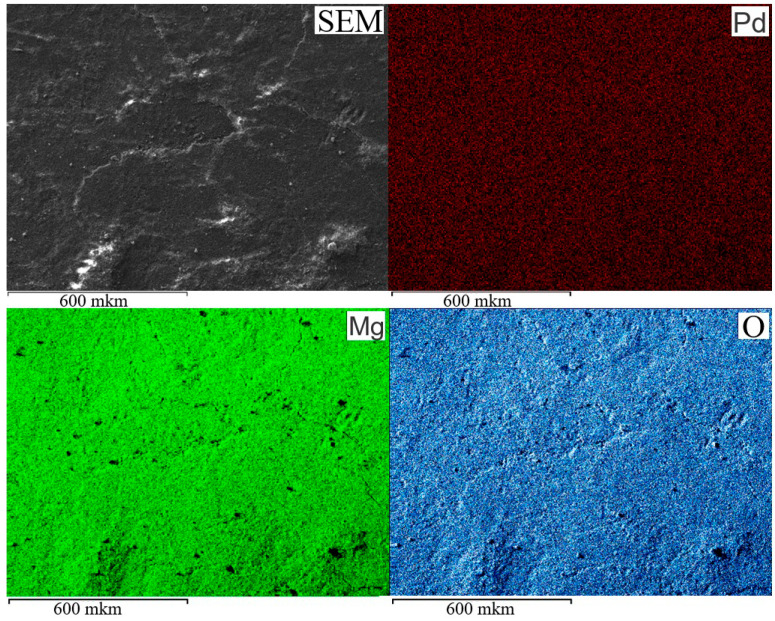
SEM/EDX elemental mapping images of 1%Pd–P4VP/MgO catalyst.

SEM and EDX elemental mapping images of Pd, Mg, and O from 1%Pd-P4VP/MgO catalyst (Figure 1) demonstrate a uniform distribution of all elements corresponding to SEM image. This suggests that Pd is homogeneously deposited on P4VP/MgO support.

**Figure 2:**
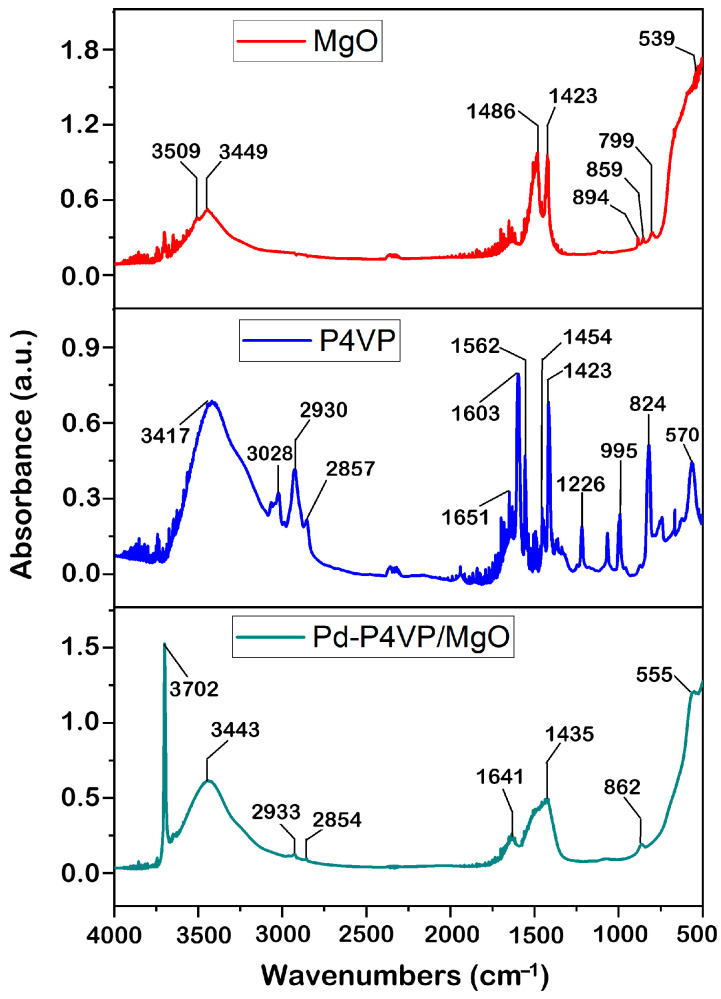
IR spectra of the MgO, P4VP, P4VP/MgO, 1%Pd–P4VP/MgO.

Figure 2 shows IR spectra of MgO, P4VP and 1%Pd–P4VP/MgO. The spectrum of MgO shows characteristic bands at 859 cm^−1^ and 894 cm^−1^ attributed to Mg–O vibrations [27,28]. The distinct bands observed at 1423 cm^−1^ and 1486 cm^−1^ are attributed to vibrations of surface hydroxyl groups [27]. The detected broad bands at 3449 cm^−1^ and 3509 cm^−1^ are due to OH stretching vibrations of water molecules [27]. In the spectrum of P4VP (Figure 2, blue line), the C=N absorption peaks are assigned at 1603 cm^−1^ and 1562 cm^−1^ due to the stretching vibration of the pyridine ring. The absorption bands at 1454 cm^−1^ and 1423 cm^−1^ correspond to stretching vibrations of C=C bond [29,30]. In the spectrum of the 1%Pd–P4VP/MgO composite, the absorption bands at 2933 and 2854 cm^−1^ are attributed to C–H stretching vibrations of the P4VP backbone, confirming the presence of the polymer. The bands observed at 1641 and 1435 cm^−1^ can be assigned to C=N and C=C stretching vibrations of the pyridine ring, although these peaks may overlap with absorption bands of –OH groups from magnesium oxide. A distinct band at 862 cm^−1^ is associated with Mg–O vibrations. Some overlap and shifting in the 1400–1650 cm^−1^ region suggest possible interactions between P4VP and MgO, as well as coordination of Pd with nitrogen atoms in the pyridine ring.

**Figure 3:**
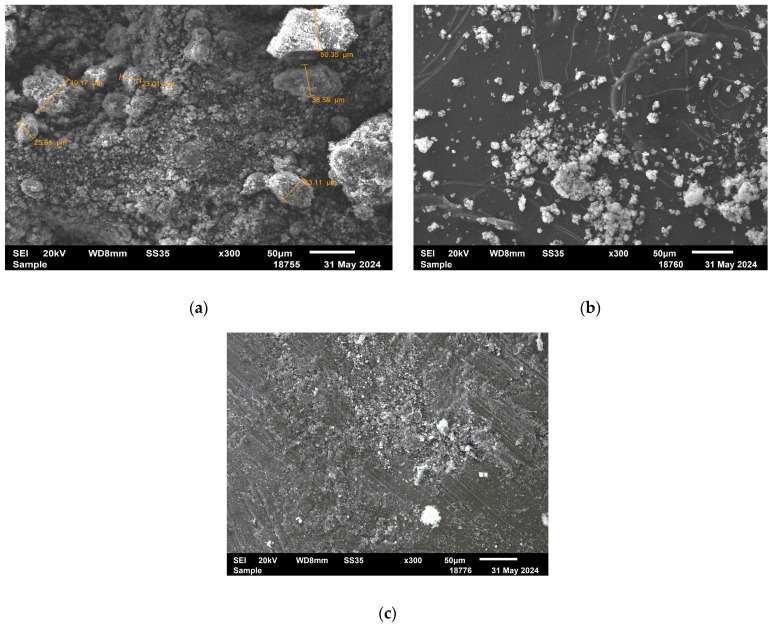
SEM images of MgO (**a**) P4VP/MgO (**b**), 1%Pd–P4VP/MgO (**c**).

Scanning electron microscopy (SEM) images reveal clear morphological changes in magnesium oxide (MgO) after modification with the polymer ligand and subsequent palladium deposition. The unmodified MgO (Figure 3a) shows a heterogeneous structure composed of both small particles and large aggregates, which appear to consist of smaller primary crystallites. In the case of the P4VP/MgO composite (Figure 3b), smaller and more compact aggregates are observed. These aggregates also appear to be composed of fine particles; however, the boundaries between them are less distinct, and the overall morphology is smoother, indicating coverage or partial embedding by the polymer. The 1%Pd–P4VP/MgO catalyst (Figure 3c) exhibits a densely packed, fused structure, likely resulting from polymer-induced agglomeration of MgO particles in the presence of palladium ions during catalyst preparation. These morphological transformations indicate successful modification of the MgO support with both the polymer and palladium species.

**Figure 4:**
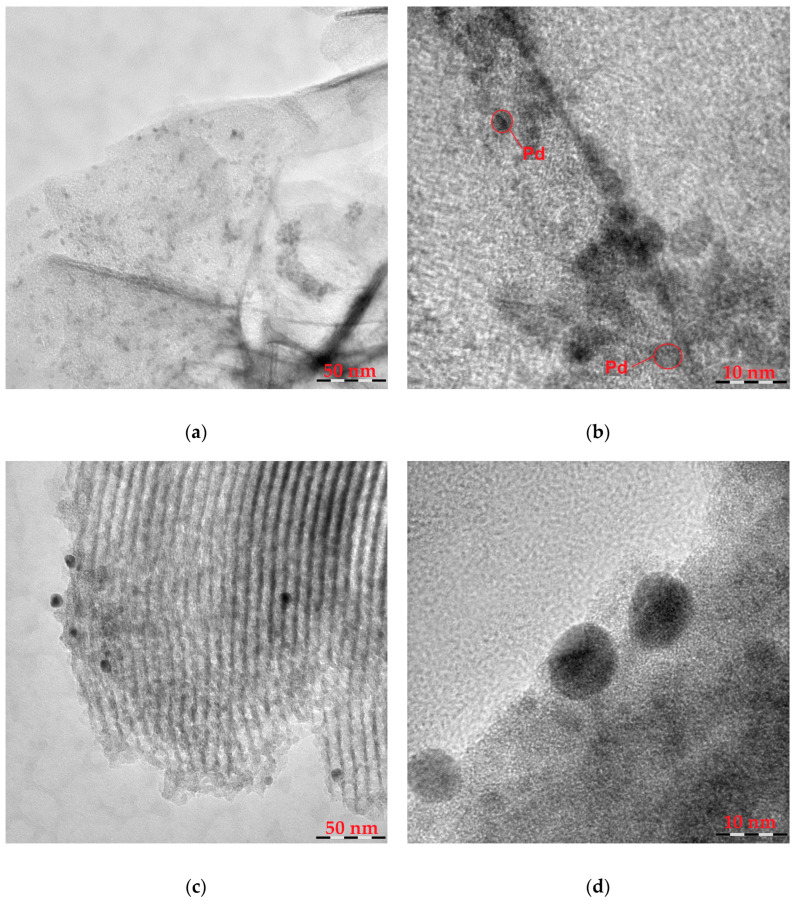
TEM images of the Pd–P4VP/MgO (**a**,**b**) and 1%Pd–P4VP/SBA-15 (**c**,**d**) catalysts at different magnifications.

TEM images of palladium catalysts immobilized on P4VP-modified MgO and SBA-15 supports are shown in Figure 4. To assess the effect of polymer inclusion on the size, shape, and distribution of palladium particles, the unmodified Pd/MgO catalyst was also examined. Before studies, the catalysts were treated with hydrogen (1 atm) in a reactor at 40 °C for 30 min. The TEM image of the 1%Pd–P4VP/MgO catalyst (Figure 4a) shows well-dispersed Pd nanoparticles uniformly distributed over the surface of the polymer-modified support. The analysis of a TEM microphotograph of the catalyst at a higher magnification level (Figure 4b) indicates that the Pd particles are approximately 2–3 nm in size. In contrast, the 1%Pd–P4VP/SBA-15 catalyst exhibits significantly larger Pd particles, with sizes ranging from 8 to 10 nm (Figure 4c,d). The image in Figure 4c also reveals uniform pore channels arranged in parallel, which is a characteristic structural feature of the SBA-15 silica matrix [21,31,32]. For the Pd/MgO catalyst, larger palladium particles (10–30 nm) were observed on the surface of the unmodified support (Figure S1).

**Figure 5:**
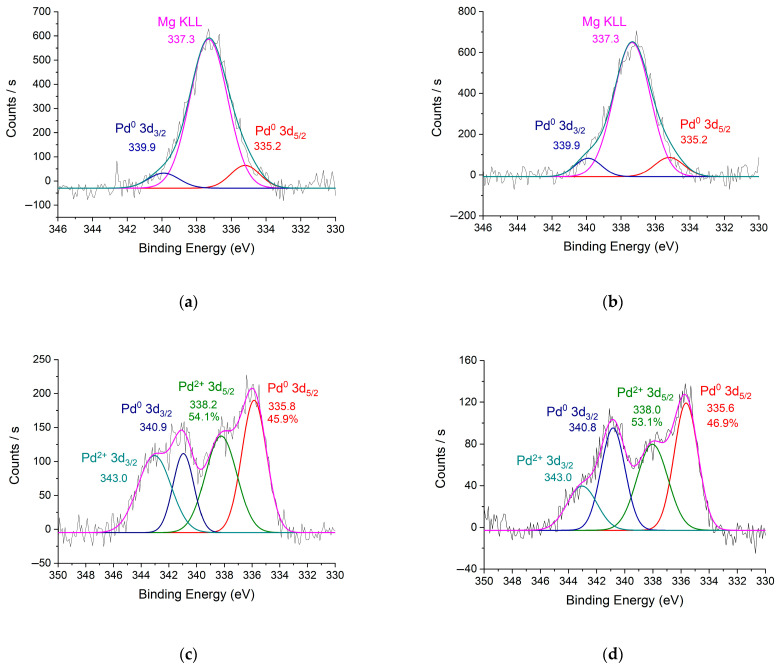
XPS spectra of Pd 3d of the 1%Pd–P4VP/MgO (**a**) and 1%Pd–CS/MgO (**b**), 1%Pd–P4VP/SBA-15 (**c**) and 1%Pd–CS/SBA-15 (**d**) after treatment with H_2_.

In order to assess the oxidation state of palladium during the hydrogenation process, the catalysts were pretreated with molecular hydrogen (1 atm) in a reactor at 40 °C for 30 min, followed by analysis using X-ray photoelectron spectroscopy (XPS). Figure 5 shows the Pd 3d region of the XPS spectra for the reduced 1%Pd–P4VP/MgO, 1%Pd–CS/MgO, 1%Pd–P4VP/SBA-15, and 1%Pd–CS/SBA-15 catalysts. XPS analysis of the 1%Pd–P4VP/MgO and 1%Pd–CS/MgO samples is complicated by the overlap of the Pd 3d signals with the Mg KLL Auger transitions, making precise interpretation difficult [33,34] (Figure 5a,b). To overcome this challenge, the spectra were processed according to the standardized methodology proposed by Major et al. [35], involving Shirley background subtraction and Voigt function-based peak fitting. Special attention was paid to the contribution of Mg KLL features in the region of interest to avoid their misinterpretation as Pd-related signals. The deconvoluted spectra revealed a shoulder at a binding energy of ~335.2 eV, which corresponds to metallic palladium (Pd^0^), as confirmed by comparison with reference values reported in the literature [36,37].

The deconvoluted Pd 3d XPS spectrum of the reduced 1%Pd–P4VP/SBA-15 catalyst revealed the presence of palladium in different oxidation states on the catalyst surface. Specifically, the Pd 3d_5_/_2_ peaks observed at 335.8 eV and 338.2 eV were assigned to metallic palladium (Pd^0^) and Pd^2+^ species, respectively [36]. The proportion of palladium in the zero-valence state was approximately 50%. A similar Pd 3d spectrum was obtained for the reduced 1%Pd–CS/SBA-15 catalyst, exhibiting nearly identical peak positions and a comparable Pd^0^ to Pd^2+^ ratio. Notably, in all cases, the Pd 3d peaks were positively shifted, which can be attributed to the stabilization of Pd species by the polymer matrices and the formation of small Pd nanoparticles [36,37].

It is worth noting that the fraction of Pd^0^ could potentially be increased by applying more extended or more intensive reduction treatments. At the same time, the presence of oxidized Pd species is not necessarily detrimental. For example, Yang et al. [38] reported a synergistic Pd-based catalyst comprising both Pd^2+^ and Pd^0^ species embedded in nitrogen-doped porous carbon (NPC), which exhibited enhanced catalytic activity in the hydrogenation of nitroarenes.

### 2.2. Catalytic Properties of Pd Catalysts, Deposited on Polymer-Modified Supports, in the Hydrogenation Process

The catalytic properties of the developed palladium catalysts were investigated in the hydrogenation of unsaturated compounds (2-propen-1-ol, phenylacetylene, 2-hexyn-1-ol) in an ethanol medium at a temperature of 40 °C and atmospheric hydrogen pressure. These mild reaction conditions were chosen based on our previous studies, where they were shown to be optimal for achieving high selectivity and activity in similar systems [12]. In addition, comparative data supporting the choice of conditions are provided in the Supplementary Information (Tables S1 and S2). The activity of catalysts was evaluated by measuring the hydrogen uptake as a function of time.

**Figure 6:**
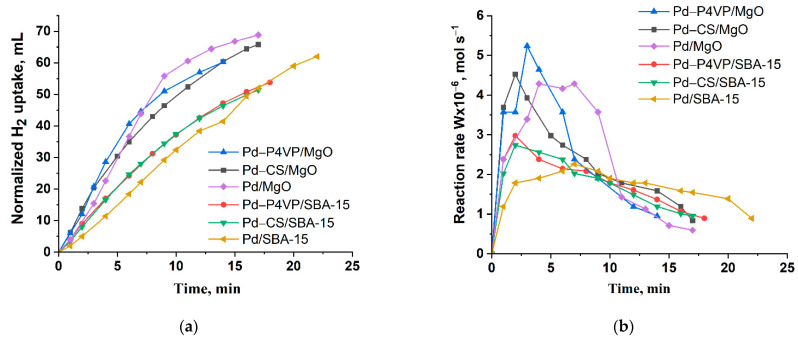
Kinetics of the hydrogen uptake (**a**) and the change in reaction rate (**b**) over the developed palladium catalysts during the hydrogenation of 2-propen-1-ol. Reaction conditions: T—40 °C, P_H2_—1 atm, m_cat_—0.05 g, solvent C_2_H_5_OH—25 mL, substrate—0.3 mL.

Figure 6 shows the results of evaluation of activity of the catalysts in hydrogenation of 2-propen-1-ol.

The semi-hydrogenation point (50 mL of H_2_ uptake) was reached after approximately 6 and 8 min of reaction for the 1%Pd–P4VP/MgO and 1%Pd–CS/MgO catalysts, respectively. In contrast, for the 1%Pd–P4VP/SBA-15 and 1%Pd–CS/SBA-15 catalysts, this value was achieved only after 15 min of reaction (Figure 6a). In the case of 1%Pd/MgO and 1%Pd/SBA-15, this value is reached after 6 and 14 min, respectively. The reaction rate profiles, calculated from the hydrogen uptake data, show that in all cases the rate initially increased sharply, reaching maximum values of 5.2 × 10^−6^, 4.5 × 10^−6^, 4.2 × 10^−6^, 3.0 × 10^−6^, 2.7 × 10^−6^, and 2.2 × 10^−6^ mol/s for 1%Pd–P4VP/MgO, 1%Pd–CS/MgO, 1%Pd/MgO, 1%Pd–P4VP/SBA-15, 1%Pd–CS/SBA-15, and 1%Pd/SBA-15, respectively, followed by a subsequent decline (Figure 6b, Table 2). The higher catalytic activity observed for the MgO-based catalysts is likely due to the smaller size of Pd nanoparticles compared to those supported on SBA-15. For example, the size of Pd particles was 2–3 nm for Pd-P4VP/MgO, compared to 8–10 nm for Pd-P4VP/SBA-15. Generally, the particle size is inversely proportional to catalytic activity, as smaller particles provide a larger surface area, which implies higher specific activity. However, despite this, Pd/MgO with larger Pd particles (10–30 nm) outperformed Pd-P4VP/SBA-15, which contained slightly smaller particles (8–10 nm). This result can be explained by the positive influence of the alkaline MgO support, which may enhance the state and performance of Pd nanoparticles in hydrogenation reactions [39]. These MgO-supported catalysts also showed higher selectivity toward propanol (80–83%) at complete substrate conversion, compared to the SBA-15-based catalysts (71–78%). Additionally, polymer-modified catalysts outperformed their unmodified counterparts in both initial activity and selectivity. Thus, 1%Pd–P4VP/MgO was identified as the most efficient catalyst in this series (Table 2).

**Figure 7:**
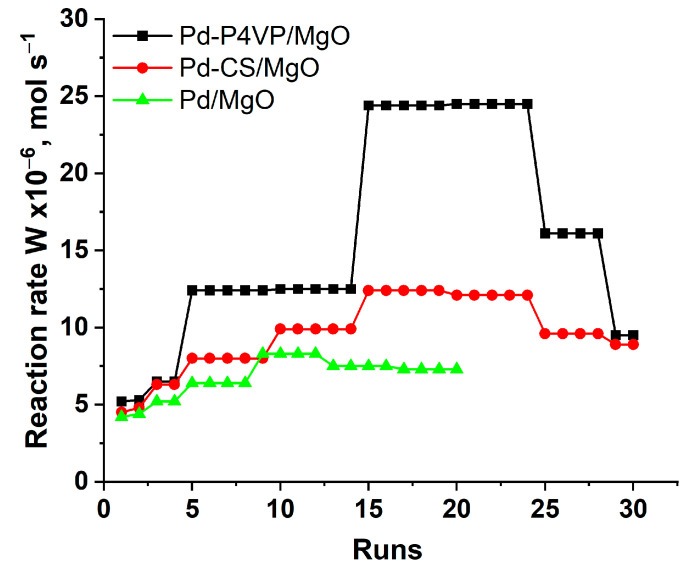
Reuse of 1% Pd–P4VP/MgO, 1% Pd–CS/MgO and 1% Pd/MgO catalysts in hydrogenation of 2-propen-l-ol.

The 1%Pd–P4VP/MgO catalyst exhibited higher activity than 1%Pd–CS/MgO and 1%Pd/MgO during the stability tests in the hydrogenation of successive portions of 2-propen-1-ol (Figure 7). Although all catalysts showed comparable activity during the first run, the differences among them increased progressively with the number of runs. For both polymer-modified systems, the reaction rate increased over the first 15 cycles, which may be attributed to the gradual swelling of the polymer–metal layer in ethanol. This effect was more pronounced for Pd–P4VP/MgO, likely due to the higher solubility and greater swelling ability of poly(4-vinylpyridine) in ethanol compared to chitosan. The slightly higher activity of the 1%Pd–CS/MgO catalyst compared to the unmodified 1%Pd/MgO also supports the beneficial role of the polymer modification in enhancing catalytic performance. After the 25th run, the activity of both polymer-modified catalysts began to decline, probably due to the accumulation of the saturated hydrogenation product, which could lead to shrinkage of the polymer layer. Nevertheless, by the 30th run, the activity of both catalysts remained higher than in the first run, confirming their overall operational stability. Similar swelling and shrinkage phenomena were reported in related polymer-modified Pd catalysts [40]. In that study, Pd–PVP/γ-Fe_2_O_3_ (PVP—polyvinylpyrrolidone) demonstrated prolonged activity due to good swelling of the PVP shell in ethanol, while Pd–Pec/γ-Fe_2_O_3_ (Pec—pectin) rapidly deactivated, likely as a result of polymer shrinkage and product accumulation. However, its catalytic performance was partially restored after washing with hot ethanol and water, supporting the proposed deactivation mechanism. This interpretation is further supported by our experimental data: according to EDX elemental analysis of the 1%Pd–P4VP/MgO catalyst after 30 hydrogenation cycles, no palladium leaching was observed. The Pd content remained unchanged at 1.0 wt.%. It should be noted, that in the case of the unmodified 1%Pd/MgO, the reaction rate also increased but less pronouncedly, which can be explained by an increasing fraction of metallic Pd^0^ in the presence of alkaline magnesium oxide during successive runs [39].

**Figure 8:**
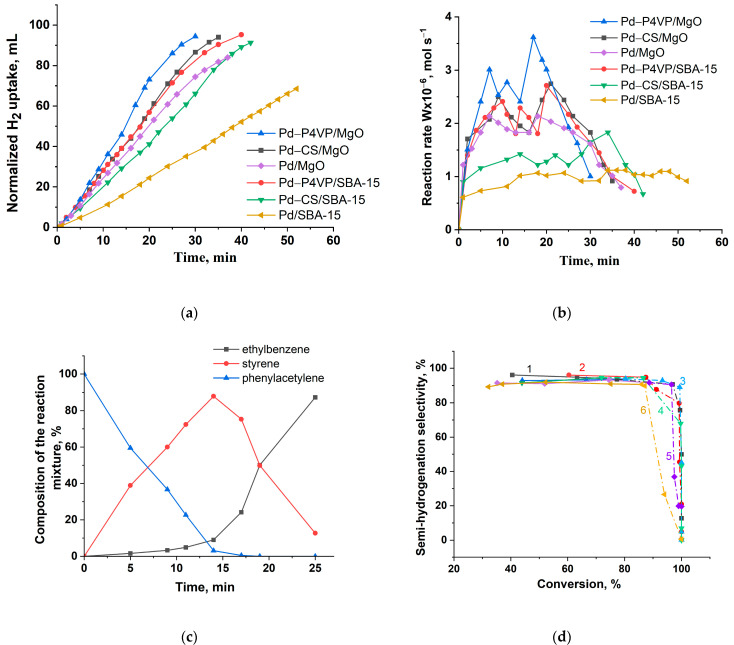
Kinetics of the hydrogen uptake (**a**), the change in reaction rate (**b**), the change in the composition of the reaction mixture on 1%Pd–P4VP/MgO catalyst (**c**) and dependence of selectivity to styrene with the substrate conversion on 1%Pd–P4VP/MgO (curve 1), 1%Pd–P4VP/SBA-15 (curve 2), 1%Pd–CS/MgO (curve 3), 1%Pd–CS/SBA-15 (curve 4), 1%Pd/MgO (curve 5) and 1%Pd/SBA-15 (curve 6) (**d**) at the hydrogenation of phenylacetylene. Reaction conditions: T—40 °C, P_H2_—1 atm, m_cat_—0.05 g, solvent C_2_H_5_OH—25 mL, substrate—0.25 mL.

The catalytic performance of P4VP-, CS-modified and unmodified palladium catalysts in the hydrogenation of phenylacetylene is shown in Figure 8. Catalysts based on MgO exhibited higher activity compared to those supported on SBA-15. At the same time, P4VP-modified catalysts demonstrated greater activity than the corresponding chitosan-containing systems. Unmodified Pd catalysts showed the lowest activity. The semi-hydrogenation point (50 mL) was reached after 14, 17, 17, 18, 28 and 38 min for 1%Pd–P4VP/MgO, 1%Pd–CS/MgO, 1%Pd–P4VP/SBA-15, 1%Pd/MgO, 1%Pd–CS/SBA-15, and 1%Pd/SBA-15, respectively (Figure 8a). The hydrogenation rate of phenylacetylene, calculated from the hydrogen uptake data, is presented in Figure 8b. In all cases, the reaction rate (W) increased rapidly within the first minutes and remained relatively constant until the semi-hydrogenation point. After that, the rate further increased, reached a maximum, and then sharply declined.

According to the chromatographic analysis, styrene is accumulated in the reaction medium on 1%Pd–P4VP/MgO in the initial period, and then is reduced to ethylbenzene (Figure 8c). Accumulation of styrene was also accompanied by the formation of small amounts of ethylbenzene, and its yield at the semi-hydrogenation point (14 min) was 9%. At the same time, the yield of styrene was 88%. The composition of the reaction mixture changed similarly on the rest Pd catalysts (Figure S2a–e). The curves of the dependence of selectivity on conversion (Figure 8d) show that all polymer-modified palladium catalysts demonstrated similar selectivity to styrene (93–95%). In contrast, the 1%Pd/MgO and 1%Pd/SBA-15 catalysts showed slightly lower selectivity (89–91%), which may be attributed to the absence of polymer on the catalyst surface. The presence of polymer likely influences the electronic properties of palladium particles, either by stabilizing them or through direct electronic interactions, thereby suppressing the further hydrogenation of styrene to ethylbenzene and enhancing overall selectivity.

The hydrogenation rate and selectivity of the catalysts during phenylacetylene hydrogenation, calculated based on hydrogen uptake and chromatographic analysis data, respectively, are summarized in Table 3.

Catalysts modified with poly(4-vinylpyridine) (P4VP) demonstrated higher activity and styrene selectivity compared to those containing chitosan (CS). Additionally, Pd catalysts supported on MgO showed greater hydrogenation activity than their SBA-15-based counterparts, as reflected in the higher hydrogenation rates for both the C≡C and C=C bonds. Notably, all polymer-modified catalysts demonstrate sufficiently high selectivity to styrene (93–95%) even at relatively high substrate conversion (77–93%). In contrast, the unmodified palladium catalysts displayed both lower activity and reduced selectivity (89–91%), likely due to the absence of stabilizing or electronically interacting polymer components on the catalyst surface.

**Figure 9:**
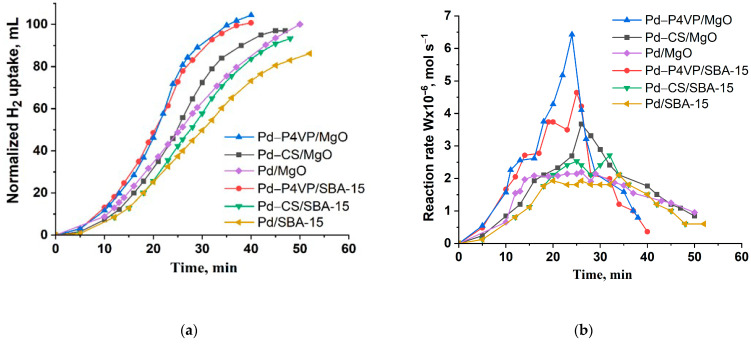

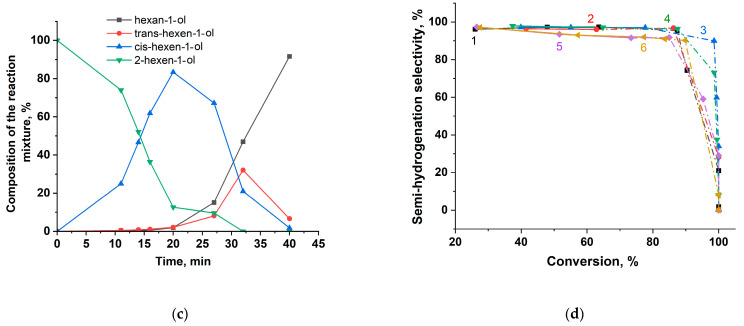
Kinetics of the hydrogen uptake (**a**), the change in reaction rate (**b**), the change in the composition of the reaction mixture on 1%Pd–P4VP/MgO catalyst (**c**) and dependence of selectivity to cis-2-hexen-1-ol with the substrate conversion on 1%Pd–P4VP/MgO (curve 1), 1%Pd–P4VP/SBA-15 (curve 2), 1%Pd–CS/MgO (curve 3), 1%Pd–CS/SBA-15 (curve 4), 1%Pd/MgO (curve 5) and 1%Pd/SBA-15 (curve 6) (**d**) at the hydrogenation of 2-hexyn-1-ol. Reaction conditions: T—40 °C, P_H2_—1 atm, m_cat_—0.05 g, solvent C_2_H_5_OH—25 mL, substrate—0.25 mL.

Figure 9 shows the results of catalyst activity evaluation in the hydrogenation of 2-hexyn-1-ol. Figure 9a presents the hydrogen uptake curves, while Figure 9b displays the reaction rate profiles calculated from the hydrogen uptake data. Catalysts modified with P4VP exhibited higher activity compared to both their chitosan-containing counterparts and the unmodified palladium catalysts. Notably, the nature of the inorganic support (MgO vs. SBA-15) had a relatively minor effect on the overall hydrogenation kinetics. However, the beneficial effect of polymer modification was more pronounced for the SBA-15-supported systems, where the difference in activity between the modified and unmodified catalysts was greater than that observed for the MgO-based samples. The semi-hydrogenation point (50 mL) was reached after 18, 24, 19, 23, 25 and 28 min for 1%Pd–P4VP/MgO, 1%Pd–CS/MgO, 1%Pd–P4VP/SBA-15, 1%Pd/MgO, 1%Pd–CS/SBA-15, and 1%Pd/SBA-15, respectively. As shown in Figure 9b, the reaction rate gradually increased during the initial stage of the reaction. After reaching the semi-hydrogenation point, the rate sharply increased, followed by a rapid drop after passing the maximum. Before the semi-hydrogenation point, both P4VP-modified catalysts (1%Pd–P4VP/MgO and 1%Pd–P4VP/SBA-15) showed comparable reaction rates. However, beyond this point, 1%Pd–P4VP/MgO demonstrated slightly higher activity. A similar trend was observed for the chitosan-based catalysts, where the MgO-supported system outperformed the SBA-15-supported one at later stages of hydrogenation. In the case of unmodified palladium catalysts, 1%Pd/MgO was more active than 1%Pd/SBA-15 throughout the entire hydrogenation process (Figure 9b).

According to the chromatographic analysis, cis-2-hexen-1-ol is accumulated in the reaction medium on 1%Pd–P4VP/MgO in the initial period, and then is reduced to hexan-1-ol (Figure 9c). Accumulation of cis-2-hexen-1-ol was accompanied by the formation of small amounts of trans-2-hexen-1-ol and hexan-1-ol, and their yield at the semi-hydrogenation point was 1.3% and 0.8%, respectively. At the same time, the yield of cis-2-hexen-1-ol was 85%. After passing the semi-hydrogenation point (18 min), part of cis-2-hexen-1-ol accumulated was also transformed to trans-2-hexen-1-ol, which was eventually reduced to hexan-1-ol. The composition of the reaction mixture changed similarly on the rest Pd catalysts (Figure S3a–e). The curves of the dependence of selectivity on conversion (Figure 9d) show that all polymer-modified catalysts demonstrated similarly high selectivity toward cis-2-hexen-1-ol (96–97%), whereas their unmodified counterparts (1%Pd/MgO and 1%Pd/SBA-15) showed noticeably lower selectivity (90–91%).

A comparison of the catalytic properties of the catalysts during the hydrogenation of 2-hexyn-1-ol is presented in Table 4.

The nature of the polymer modifier significantly affected the catalysts activity. Catalysts modified with P4VP were more active than those containing chitosan, while both types of polymer-modified systems outperformed the unmodified palladium catalysts. In contrast, the choice of support (MgO or SBA-15) had almost no effect on the rate of triple bond (C≡C) hydrogenation, but slightly influenced the hydrogenation of the double bond (C=C), with MgO-based catalysts being slightly more active than those supported on SBA-15 at this stage. It is noteworthy that polymer modification led to increased selectivity toward cis-2-hexen-1-ol compared to the corresponding unmodified catalysts. Both P4VP- and CS-modified systems demonstrated higher selectivity (96–97%), while the unmodified catalysts showed noticeably lower values (90–91%).

Thus, catalytic testing in the hydrogenation of three different substrates demonstrated that polymer modification is a promising strategy for enhancing both the activity and selectivity of supported palladium catalysts. Among the tested systems, catalysts modified with P4VP showed superior performance, which can be attributed to the good swelling ability of P4VP in ethanol (the solvent used in all three reactions). The nature of the inorganic support also influenced catalytic behavior, with MgO-based systems exhibiting higher activity than their SBA-15 counterparts. This difference can be explained by the smaller palladium particle sizes observed in MgO-supported catalysts, which likely contribute to improved catalytic performance. Overall, the 1%Pd–P4VP/MgO catalyst exhibited the highest activity and selectivity across all three hydrogenation reactions, making it the most efficient system among those investigated. Notably, the catalytic properties of the developed 1%Pd–P4VP/MgO catalyst were comparable to those of other well-known Pd-based catalysts reported in the literature (Table 5).

## 3. Materials and Methods

### 3.1. Materials

Chitosan (CS, Mw 250,000), poly(4-vinylpyridine) (P4VP, Mw 60,000), magnesium oxide (MgO, pure grade), mesostructured silica SBA-15, palladium(II) chloride (PdCl_2_, Pd content 59–60%), 2-propen-1-ol, phenylacetylene and 2-hexyn-1-ol were purchased from Sigma-Aldrich, St. Louis, MO, USA. Ethanol (reagent grade) was obtained from Talgar Alcohol LLP (Talgar, Kazakhstan).

### 3.2. Synthesis of Pd Catalysts

The catalysts were prepared via a sequential adsorption method [12,47]. A 5 mL solution of 1.9 × 10^−2^ M P4VP (0.01 g in 5 mL of water) or CS (0.016 g in 5 mL of water) was added dropwise to a suspension of the support (1 g MgO or SBA-15 in 15 mL of water) at room temperature, and resulting mixture was stirred for 2 h. Then, 5 mL of a 1.9 × 10^−2^ M K_2_PdCl_4_ solution was added dropwise and stirred for 3 h. The concentration of polymer and palladium salt solutions (1.9 × 10^−2^ M) were taken from a calculation for the preparation of catalysts with 1% palladium content and [Pd: polymer] molar ratio = 1:1 [47]. The resulting catalyst was kept in the mother liquor for 12–15 h. Thereafter, it was washed with water, and then dried in air. For comparison, unmodified Pd/MgO and Pd/SBA-15 catalysts were prepared using the same procedure, except that no polymer was added.

The quantitative content of Pd in aqueous solutions was detected via photoelectrocolorimetry (PEC). The measurement was carried out using an SF-2000 UV/Vis spectrophotometer (OKB Spektr, St. Petersburg, Russia) according to calibration curves (wavelength λ = 425 nm) [23].

### 3.3. Characterization of the Catalysts by Physicochemical Methods

Scanning electron microscopy (SEM) micrographs were obtained on a scanning electron microscope JSM-6610 LV (“JEOL” Ltd., Tokyo, Japan) at an accelerating voltage of 15–20 kV. EDX elemental analysis was performed using an energy-dispersive detector built into the microscope (EDX Oxford Instruments, Oxford, UK) [12].

IR spectra were obtained using a Nicolet iS5 from Thermo Scientific (Waltham, MA, USA), with a resolution of 3 cm^−1^ in the 4000–400 cm^−1^ region. Pellets for infrared analysis were obtained by grinding a mixture of 1 mg sample with 100 mg dry KBr, followed by pressing the mixture into a mold [12].

Transmission electron microscopy (TEM) micrographs were obtained on a Zeiss Libra 200FE transmission electron microscope (Carl Zeiss, Oberkochen, Germany) with an accelerating voltage of 100 kV [12].

X-ray photoelectron spectra (XPS) of palladium composites were recorded on an ESCALAB 250Xi X-ray and ultraviolet photoelectron spectrometer (Thermo Fisher Scientific, Waltham, MA, USA) [12].

### 3.4. Methodology of Hydrogenation

The hydrogenation of the 2-propen-1-ol and acetylene compounds (2-hexyn-1-ol and phenylacetylene) was carried out in a thermostated glass reactor according to the procedure described in Ref. [13]. The hydrogenation of unsaturated compounds was carried out with H_2_ from a gas storage burette connected to the reactor in an ethanol medium (25 mL) at atmospheric hydrogen pressure and a temperature of 40 °C, under intensive stirring (600–700 oscillations per minute) [40]. Before hydrogenation, the nanocatalyst (0.05 g) was reduced with hydrogen (1 atm) in a reactor at 40 °C for 30 min under conditions of intensive stirring. After the hydrogen treatment, a substrate (0.3 mL of 2-propen-1-ol and 0.25 mL of 2-hexyn-1-ol and phenylacetylene) was added to the reactor.

The hydrogenation products were analyzed using gas–liquid chromatography on a Chromos GC1000 chromatograph (Chromos Engineering, Dzerzhinsk, Russia) with a flame ionization detector in isothermal mode. A BP21 capillary column (FFAP) with a polar phase (PEG modified with nitroterephthalate) was used. This device was 50 m in length and 0.32 mm in inner diameter. The column temperature was 90 °C, the injector temperature was 200 °C, and helium served as the carrier gas. A total of 0.2 mL of the sample was investigated. Selectivity was calculated as the fraction of the target product present in the reaction products at a given degree of substrate conversion [13].

## 4. Conclusions

The primary goal of this study was to investigate how the nature of the polymer modifier and the inorganic support influences the structure and catalytic behavior of Pd-based hybrid catalysts in the selective hydrogenation of unsaturated compounds. To this end, a series of Pd-based catalysts supported on MgO and mesostructured silica SBA-15, modified with P4VP and CS, were successfully synthesized via sequential adsorption of polymers and palladium ions under ambient conditions. To evaluate the influence of the polymer on the catalytic properties of palladium-based systems, catalysts without polymer modification (Pd/MgO and Pd/SBA-15) were also synthesized. Comprehensive physicochemical characterization using spectrophotometry, elemental analysis, IR spectroscopy, SEM, TEM, and XPS confirmed the formation of ternary hybrid systems (metal–polymer–support), with strong evidence of metal anchoring and potential interactions between the polymer and support surface.

TEM analysis revealed that the nature of the inorganic support plays a crucial role in determining Pd nanoparticle size and dispersion. Pd–P4VP/MgO catalysts showed uniformly distributed nanoparticles with smaller sizes (2–3 nm), while Pd–P4VP/SBA-15 systems exhibited larger, less uniformly distributed particles (8–10 nm). Additionally, the presence of the polymer itself had a pronounced effect on palladium dispersion. The unmodified Pd/MgO catalyst exhibited significantly larger palladium particles (10–30 nm) on the support surface. XPS data obtained after hydrogen pretreatment of the catalysts revealed partial reduction of Pd^2+^ to Pd^0^ (~50%), indicating that during hydrogenation conditions, palladium exists in a mixed oxidation state. All samples exhibited binding energy shifts, which may be attributed to metal–polymer interactions and the small size of Pd nanoparticles.

Analysis of the hydrogenation results obtained for three model substrates (2-propen-1-ol, phenylacetylene, and 2-hexyn-1-ol) under mild conditions (40 °C, 1 atm H_2_) revealed the following key observations:

− Polymer-containing catalysts showed improved activity and selectivity compared to unmodified ones, confirming that polymer modification has a positive effect on the performance of palladium-based catalysts. This improvement is likely associated with the stabilizing effect of the polymer, which promotes the formation of smaller and more uniformly distributed palladium nanoparticles, thereby enhancing catalytic efficiency.
− Catalysts supported on MgO exhibited higher activity in the hydrogenation of 2-propen-1-ol and phenylacetylene compared to those based on SBA-15, which correlates with the formation of smaller and more uniformly dispersed Pd nanoparticles (2–3 nm on MgO vs. 8–10 nm on SBA-15), as confirmed by TEM analysis. In contrast, for the hydrogenation of 2-hexyn-1-ol, the nature of the inorganic support had an insignificant effect on catalytic activity.
− P4VP-modified catalysts demonstrated higher activity than their chitosan-containing counterparts across all studied hydrogenation reactions. This difference became particularly pronounced during long-term stability tests conducted for Pd–P4VP/MgO and Pd–CS/MgO in the hydrogenation of 2-propen-1-ol. In both cases, the reaction rate increased progressively with each subsequent substrate addition, likely due to swelling of the polymer layer in ethanol. However, the rate enhancement was more significant for the Pd–P4VP/MgO system, which can be attributed to the superior swelling capacity of poly(4-vinylpyridine) compared to chitosan. The chitosan-modified catalyst (1%Pd–CS/MgO) also outperformed the unmodified 1%Pd/MgO system, confirming the beneficial effect of polymer modification. In contrast, the unmodified catalyst showed only a slight increase in activity during repeated cycles, which can be explained by the gradual increase in the fraction of metallic palladium (Pd^0^) in the presence of alkaline magnesium oxide after each successive cycle.
− Polymer modification noticeably increased the selectivity of catalysts toward the target products in all three hydrogenation reactions, which can be explained by the influence of the polymer on the electronic state of palladium. For example, the selectivity to propanol in the hydrogenation of 2-propen-1-ol was higher for polymer-modified catalysts (77–83%) compared to unmodified ones (71–80%). Similarly, in phenylacetylene hydrogenation, the selectivity to styrene for unmodified catalysts was 89–91%, while polymer-modified catalysts achieved 93–95%. In the case of 2-hexyn-1-ol hydrogenation, unmodified catalysts showed selectivity to cis-2-hexen-1-ol of 90–91%, compared to 96–97% for polymer-modified systems. For polymer-modified catalysts, selectivity trends were found to be substrate-dependent. In the hydrogenation of 2-propen-1-ol, MgO-supported catalysts exhibited higher selectivity toward propanol than their SBA-15-supported counterparts. In the case of 2-hexyn-1-ol and phenylacetylene hydrogenation, neither the type of support nor the nature of the polymer modifier significantly affected selectivity: all catalysts exhibited consistently high selectivity to cis-2-hexen-1-ol (96–97%) and styrene (93–95%), respectively.

These findings demonstrate that by varying the nature of the polymer modifier and the inorganic support, it is possible to tune both the activity and selectivity of polymer-modified palladium catalysts for selective hydrogenation reactions. The hybrid approach combining metal, polymer, and support opens up promising pathways for designing efficient and tunable heterogeneous catalytic systems. Importantly, the results highlight that the optimal choice of polymer and support should be made individually for each target reaction, as their influence on catalytic performance is strongly substrate-dependent.

Although the present study was conducted under laboratory-scale batch conditions, several factors suggest the potential applicability of the developed Pd–P4VP/MgO catalysts on a larger scale. First, the catalyst exhibits high activity, selectivity, and excellent stability over multiple hydrogenation cycles, without Pd leaching. These features are essential for practical industrial use, where catalyst durability and metal retention are critical for cost-effectiveness and regulatory compliance. Second, the synthesis of the catalyst involves inexpensive and readily available materials, such as MgO and P4VP, and the reactions are performed in ethanol, a relatively green and industrially acceptable solvent. This makes the overall system attractive from both economic and environmental perspectives.

## Figures and Tables

**Table 1:** Results of Pd ions deposition on polymer-modified support.

Catalyst	m(Pd) Before Adsorption, Mg	m(Pd) After Adsorption, Mg	Ads. Degree, %	Pd Content, %	
				PEC	EDX
1%Pd–P4VP/MgO	10.1	0.1	99	1.0	1.0
1%Pd–P4VP/MgO (after 30 cycles)		-	-	-	1.0
1%Pd–CS/MgO		0.1	99	1.0	1.0
1%Pd–P4VP/SBA-15		0.4	96	1.0	n.a.*
1%Pd–CS/SBA-15		0.5	95	1.0	n.a.*
1%Pd/MgO		0.5	95	1.0	1.0
1%Pd/SBA-15		0.5	95	1.0	1.0

n.a.*—not analyzed.

**Table 2:** Catalytic properties of Pd catalysts in the hydrogenation of 2-propen-1-ol *.

Catalyst	W_max_·10^−6^, mol s^−1^	Selectivity, %		Conversion, %
		Propanal	Propanol	
1%Pd–P4VP/MgO	5.2	16.6	83.4	100
1%Pd–CS/MgO	4.5	17.8	82.2	100
1%Pd/MgO	4.2	19.2	80.2	100
1%Pd–P4VP/SBA-15	3.0	22.6	77.4	100
1%Pd–CS/SBA-15	2.7	22.0	78.0	100
1%Pd/SBA-15	2.2	14.0	71.0	100

* Experimental conditions: T—40 °C, P_H2_—1 atm, m_cat_—0.05 g, solvent C_2_H_5_OH—25 mL, substrate—0.3 mL.

**Table 3:** Results of the hydrogenation of phenylacetylene on synthesized Pd catalysts *.

Catalyst	W_max_·10^−6^, mol s^−1^		Selectivity to Styrene, %	Conversion, %
	C≡C	C=C		
1%Pd–P4VP/MgO	3.0	3.6	94	77
1%Pd–CS/MgO	2.5	2.7	93	93
1%Pd/MgO	1.9	2.1	91	88
1%Pd–P4VP/SBA-15	2.4	2.7	95	88
1%Pd–CS/SBA-15	1.3	1.8	94	87
1%Pd/SBA-15	1.1	1.2	89	87

* Experimental conditions: T—40 °C, P_H2_—1 atm, m_cat_—0.05 g, solvent C_2_H_5_OH—25 mL, substrate—0.25 mL.

**Table 4:** The results of 2-hexyn-1-ol hydrogenation on the synthesized Pd catalysts *.

Catalyst	W·10^−6^, mol s^−1^		Selectivity to cis-2-hexen-1-ol, %	Conversion, %
	C≡C	C=C		
1%Pd–P4VP/MgO	3.7	6.4	97	81
1%Pd–CS/MgO	2.7	3.7	97	78
1%Pd/MgO	2.1	2.3	91	80
1%Pd–P4VP/SBA-15	3.7	4.6	97	86
1%Pd–CS/SBA-15	2.5	2.7	96	88
1%Pd/SBA-15	1.9	2.1	90	89

* Experimental conditions: T—40 °C, P_H2_—1 atm, m_cat_—0.05 g, solvent C_2_H_5_OH—25 mL, substrate—0.25 mL.

**Table 5:** A comparison of catalytic properties of the 1%Pd–P4VP/MgO with other Pd catalysts in hydrogenation of alkynes and alkynols.

Catalyst	Substrate	T, °C	Pressure, MPa	TOF, s^−1^	Selectivity, %	Ref.
1%Pd–P4VP/MgO	2-hexyn-1-ol	40	0.1	0.7	97	This study
Pd@TiO_2_CA	2-hexyn-1-ol	40	0.1	1.2	96	[40]
Pd/FDU-12(4.5)_CAB_0.02	3-hexyn-1-ol	35	0.3	-	95	[41]
Pd@NKZPD	3-hexyn-1-ol	r.t.*	1.0	0.4	55	[42]
1%Pd–P4VP/MgO	phenylacetylene	40	0.1	0.6	94	This study
HHTPTA-Pd	phenylacetylene	40	0.1	-	97	[43]
Pd/Ni@G	phenylacetylene	30	0.2	2.0	93	[44]
Pd-PVP/MNPs	phenylacetylene	40	0.1	0.9	94	[45]
Fe_3_O_4_@ZIF-8/Pd	phenylacetylene	40	0.1	0.5	93	[46]

r.t.*—room temperature.

## Data Availability

The data that support the findings of this study are available from the corresponding author upon reasonable request.

## Supplementary Materials

The following supporting information can be downloaded at: https://www.mdpi.com/article/10.3390/molecules30183820/s1, Figure S1: TEM image of the 1%Pd/MgO catalyst; Figure S2: Changes in the composition of the reaction mixture during the hydrogenation of phenylacetylene in the presence of 1%Pd–CS/MgO (a), 1%Pd–P4VP/SBA-15 (b), 1%Pd–CS/SBA-15 (c), 1%Pd/MgO (d) and 1%Pd/SBA-15 (e); Figure S3: Changes in the composition of the reaction mixture during the hydrogenation of 2-hexyn-1-ol in the presence of 1%Pd–CS/MgO (a), 1%Pd–P4VP/SBA-15 (b), 1%Pd–CS/SBA-15 (c), 1%Pd/MgO (d) and 1%Pd/SBA-15 (e); Table S1: Effect of variations in temperature on catalytic performance of 1%Pd–P4VP/MgO catalyst in the 2-propen-1-ol hydrogenation; Table S2: Effect of variations in catalyst dosage on catalytic performance of 1%Pd–P4VP/MgO catalyst in the 2-propen-1-ol hydrogenation.
