# Supplementary material for: Effect of Support and Polymer Modifier on the Catalytic Performance of Supported Palladium Catalysts in Hydrogenation
Source: Molecules. 2025 Sep 19;30(18):3820. doi: 10.3390/molecules30183820 (PMC12472476; doi:10.3390/molecules30183820)
Supplement: Supplementary file 1 [file molecules-30-03820-s001.zip › molecules-3857483-supplementary.pdf]

Supplementary Information for:

Effect of Support and Polymer Modifier on the Catalytic Performance of Supported Palladium Catalysts in Hydrogenation

Assemgul S. Auyezkhanova *, Eldar T. Talgatov, Sandugash N. Akhmetova, Aigul I. Jumekeyeva, Akzhol A. Naizabayev, Aigul T. Zamanbekova and Makpal K. Malgazhdarova

D.V. Sokolsky Institute of Fuel, Catalysis, and Electrochemistry, Kunaev Str. 142, Almaty 050010, Kazakhstan; e.talgatov@ifce.kz (E.T.T.); s.akhmetova@ifce.kz (S.N.A.); a.dzhumekeeva@ifce.kz (A.I.J.); a.naizabayev@ifce.kz (A.A.N.); a.zamanbekova@ifce.kz (A.T.Z.); m.malgazhdarova@ifce.kz (M.K.M.)

* Correspondence: a.auezkhanova@ifce.kz

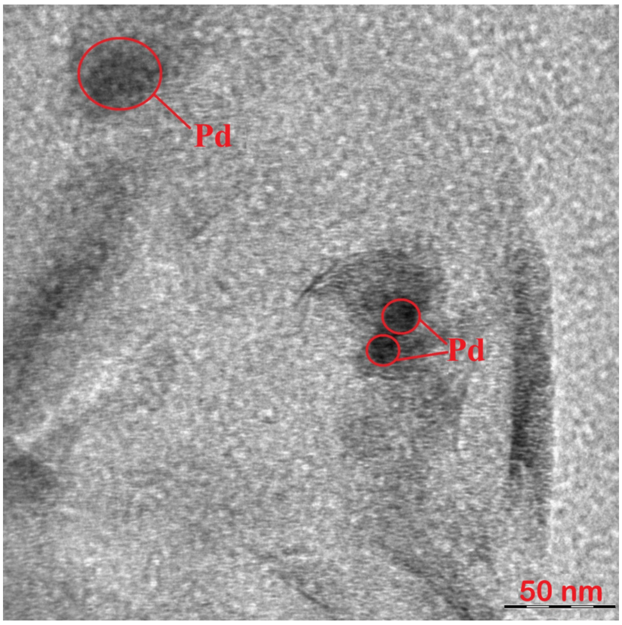

Figure. S1. TEM image of the 1%Pd/MgO catalyst.

Table S1. Effect of variations in temperature on catalytic performance of 1%Pd–P4VP/MgO catalyst in the 2-propen-1-ol hydrogenation *.

Catalyst	Temperature, °C	$W_{max}\cdot 10^{-6}$, mol s^{-1}	Selectivity, %		Conversion, %
			Propanal	Propanol	
1%Pd–P4VP/MgO	20	1.6	21.0	79.0	100
	30	1.9	19.8	80.2	100
	40	5.2	16.6	83.4	100
	50	2.0	23.4	76.6	100

* Experimental conditions: T—20 °C, 30 °C, 40 °C, 50 °C, P_{H2}—1 atm, m_{cat}—0.05 g, solvent C_2H_5OH—25 mL, substrate—0.3 mL.

Table S2. Effect of variations in catalyst dosage on catalytic performance of 1%Pd–P4VP/MgO catalyst in the 2-propen-1-ol hydrogenation *.

Catalyst	Catalyst Dosage, g	$W_{max}\cdot 10^{-6}$, mol s^{-1}	Selectivity, %		Conversion, %
			Propanal	Propanol	
1%Pd–P4VP/MgO	0.01	1.8	26.8	73.2	100
	0.03	2.9	20.8	79.2	100
	0.05	5.2	16.6	83,4	100
	0.1	4.7	18.2	81.8	100

* Experimental conditions: T—40 °C, P_{H2}—1 atm, m_{cat}—0.01 g, 0.03 g, 0.05 g 0.1 g, solvent C_2H_5OH—25 mL, substrate—1.0 mL.

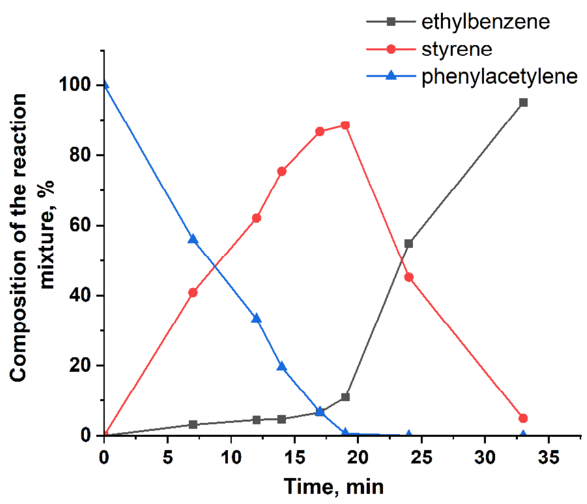
ethylbenzene
styrene
phenylacetylene
Composition of the reaction mixture, %
Time, min

(a)

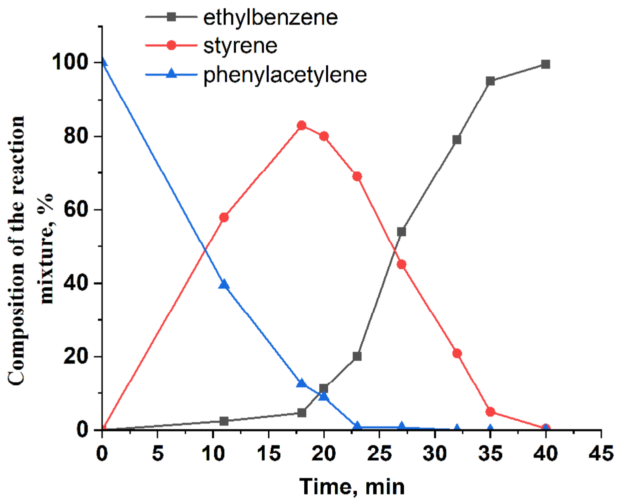
ethylbenzene
styrene
phenylacetylene
Composition of the reaction mixture, %
Time, min

(b)

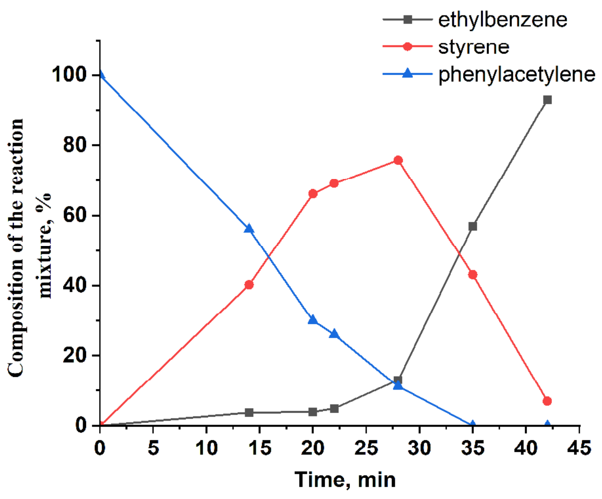
ethylbenzene
styrene
phenylacetylene
Composition of the reaction mixture, %
Time, min

(c)

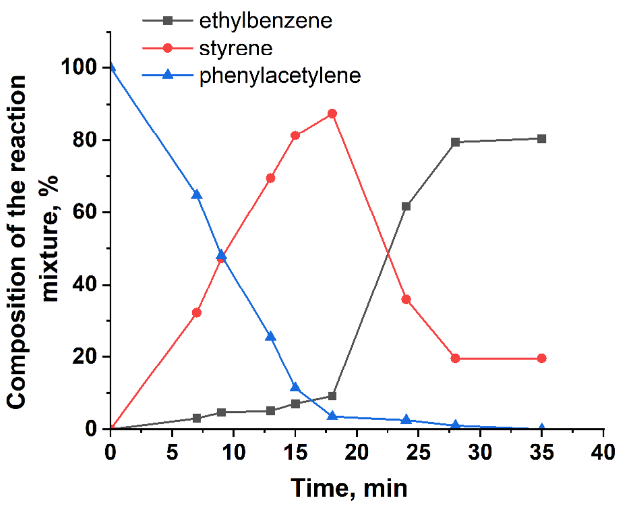
ethylbenzene
styrene
phenylacetylene
Composition of the reaction mixture, %
Time, min

(d)

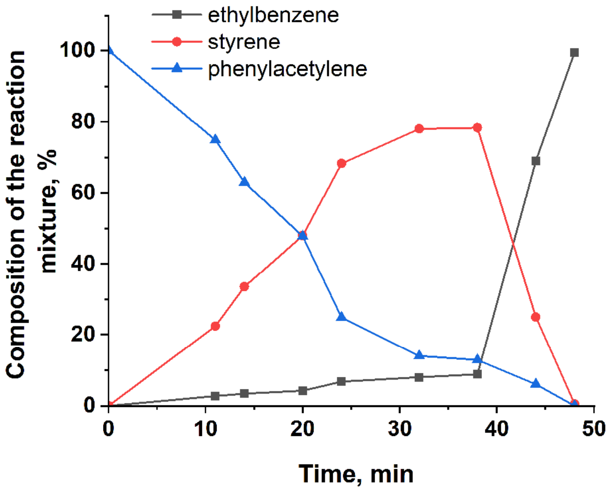

(e)

Figure. S2. Changes in the composition of the reaction mixture during the hydrogenation of phenylacetylene in the presence of 1%Pd–CS/MgO (**a**), 1%Pd–P4VP/SBA-15 (**b**), 1%Pd–CS/SBA-15 (**c**), 1%Pd/MgO (**d**) and 1%Pd/SBA-15 (**e**). Reaction conditions: T—40 °C, P_{H2}—1 atm, m_{cat}—0.05 g, solvent C_2H_5OH—25 mL, substrate—0.25 mL.

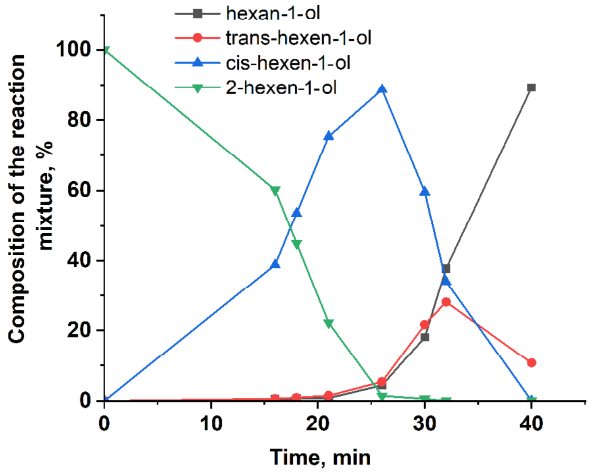

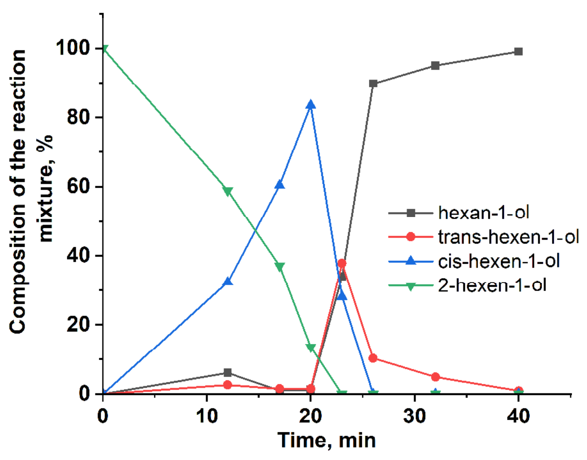

(a) **(b)**

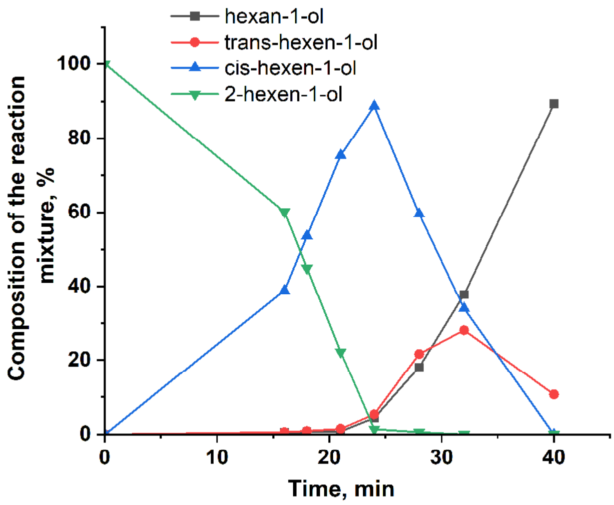

(c)

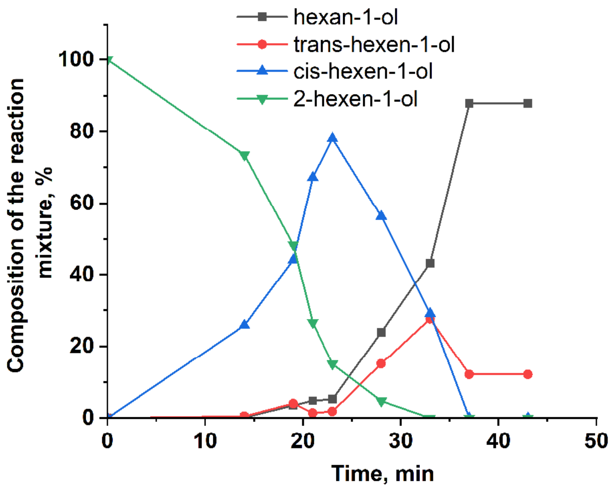

(d)

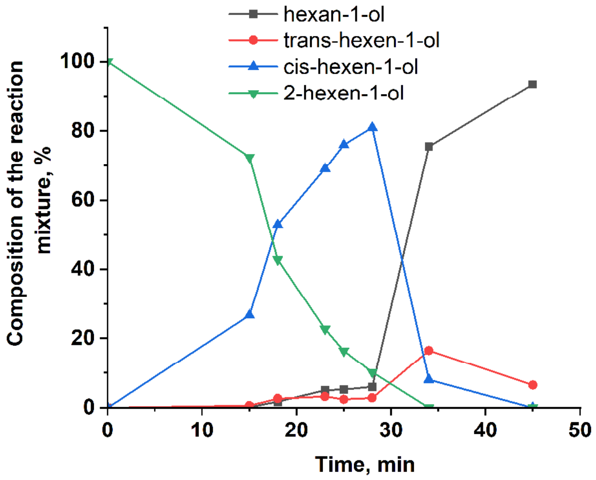

(e)

Figure. S3. Changes in the composition of the reaction mixture during the hydrogenation of 2-hexyn-1-ol in the presence of 1%Pd–CS/MgO (**a**), 1%Pd–P4VP/SBA-15 (**b**), 1%Pd–CS/SBA-15 (**c**), 1%Pd/MgO (**d**) and 1%Pd/SBA-15 (**e**). Reaction conditions: T—40°C, P_{H2}—1 atm, m_{cat}—0.05 g, solvent C_2H_5OH—25 mL, substrate—0.25 mL.
